# Transplant Versus Non-Transplant Hepatocellular Carcinoma Patient Characteristics And Survival

**DOI:** 10.52338/wjoncgy.2025.4616

**Published:** 2025-04-10

**Authors:** Brian I. Carr, Volkan Ince, Harika Gozukara Bag, Ramazan Kutlu, Sezai Yilmaz

**Affiliations:** 1 Liver Transplant Institute, Inonu University Faculty of Medicine, 44280, Malatya, Turkiye.; 2 Department of Surgery, Inonu University Faculty of Medicine, 44280, Malatya, Turkiye.; 3 Department of Biostatistics, Inonu University Faculty of Medicine, 44280, Malatya, Turkiye.; 4 Department of Radiology, Inonu University Faculty of Medicine, 44280, Malatya, Turkiye

**Keywords:** HCC, transplant, size, survival, cirrhosis

## Abstract

**Background and Objectives:**

Survival of patients treated by liver transplantation for hepatocellular carcinoma (HCC) remains excellent, with more that 75% 5-year survival. By contrast, overall survival of HCC patients in large populations remains less than 30% at 5 years. Our aims were to examine whether this discrepancy was due to the low proportion of patients who get treated by liver transplant in our HCC cohort and why.

**Materials and Methods:**

New patients presenting with HCC at our institution over the last 5 years were evaluated in this prospective study. Baseline tumor evaluation was done by CAT scan and routine hematology and liver function laboratory values were recorded, as was survival.

**Results:**

Almost all new HCC patients (n=628) over 5 years at Inonu University hospital were evaluated. 191 patients (30.4% of the total cohort) received potentially curative and survival-extending liver transplants, while 384 patients (61.1% of the total cohort) received non-surgical therapies, 53 patients (8.4%) could not receive any oncologic therapy.

Transplanted HCC patients had smaller, less aggressive HCCs, worse liver function and a mean survival of 43.06 + 1.41 months. Non-surgically treated HCC patients had larger, more aggressive HCCs, better liver function, and a mean survival of 31.51±1.53 months, p<0.001. No-therapy patients had both most aggressive HCCs and worst liver function, and a mean survival of 4.41±0.95 months.

**Conclusions:**

Survival after liver transplant was significantly longer than without liver transplant. Future efforts need to focus on HCC prevention, early detection, and in identifying/treating additional HCC patients who could be rendered transplant-eligible.

**Lay summary.:**

Long survival is mainly associated with liver transplant, yet only one third of our patients were eligible for transplant, because the other patients had tumors that were too advanced for transplantation at presentation.

**Simple Summary:**

Hepatocellular carcinoma (HCC) is essentially cured (>75% 5-years survival) when patients within accepted criteria are treated by liver transplant. Other, non-surgical treatments result in dramatically shorter survival times. We collected data on all patients being referred for HCC treatment over a 5-year period and found that only 30.4% were offered transplant. To investigate the reasons, we compared baseline clinical and tumor characteristics of all new patients on presentation at our institute and found that transplanted patients have smaller and less aggressive HCCs. We discuss whether this is due to a different HCC biology or absence of surveillance or whether the transplant criteria might be too stringent.

## INTRODUCTION

Multiple new oncological treatments have been introduced into clinical practice in the last 20 years for the treatment of patients with advanced stages of hepatocellular carcinoma (HCC), who are ineligible for potentially curative liver transplantation. These agents include transhepatic arterial radioembolization (TARE), the tyrosine kinase inhibitors sorafenib, regorafenib, lenvatinib, ramucirumab and cabozantinib [1], as well as the immune checkpoint inhibitors, either in combination with an anti-angiogenic agent (atezolizumab plus bevacizumab) or the combination of 2 different classes of immune checkpoint inhibitor (durvalumab plus tremelimumab) [2, 3]. This combination recently showed a 48-month overall survival rate of 25.2% [3, 4]. Although we do not yet have approval for use of immune checkpoint inhibitor drugs for HCC in Turkiye, we wished to evaluate the percentage and survival of patients selected for live-donor liver transplant versus any other therapy, in our high HCC throughput institution. This was made possible by the identification and documentation over the last 5 years of almost all HCC patients presenting at our institute and recorded in our weekly liver cancer tumor board, including all HCC patients selected for liver transplant, loco-regional therapy, medical oncology referral, or no therapy/best supportive care [BSC]. We show here that in the pre-immune checkpoint inhibitor period, no non-surgical therapy came close to the survival resulting from liver transplant for HCC patients. This may reflect selection of patients for liver transplant who have less aggressive tumor biology, but also points to the low percent of patients who are offered potentially curative therapy due to their advanced stage at presentation.

## METHODS

### Clinical

This prospective study was based upon the patients presented to our weekly institutional Liver Tumor Board from 2019–2023. Almost all patients presenting with HCC at our institution over the last 5 years were evaluated in this prospective study. Baseline tumor evaluation was done by computerized axial tomography (CAT) scan assessment of maximum tumor diameter (MTD) and tumor numbers. Routine hematology and liver function and alpha-fetoprotein (AFP) values were recorded, as were demographics and survival.

#### Statistical.

Normality of the quantitative data was assessed by Shapiro-Wilk test. Two independent group comparisons were performed by Mann-Whitney U test. Median, interquartile range, minimum and maximum values were used to summarize the quantitative data. Distribution of the qualitative data was presented by count and percentage. Comparisons according to qualitative data were performed by continuity-corrected chi-square test or Pearson’s chi-square test due to sample size assumptions. Kaplan-Meier method was used for survival estimations and Log-Rank test was used for survival comparison between groups. Cox regression was used to obtain Hazard Ratios. In all analyses two-way significance level considered to be <0.05.

### Ethical considerations

Patient information was prospectively collected and de-identified. This study was approved by the Inonu University scientific research and publication ethics board, Health Sciences Non-Interventional Clinical Research Ethics Board, approval decision #2024/6196. This work complied with the guidelines for human studies and was conducted ethically in accordance with the World medical Association Declaration of Helsinki.

## RESULTS

### Total cohort

Almost all new HCC patients (including 191 for live donor liver transplantation, 384 for non-surgical oncologic therapies and 53 receiving best supportive care, total 628) presenting over 5 years at Inonu University hospital, were evaluated. Characteristics of the unstratified total cohort and survival is shown in Tables 1 and 2. The median age was 61 years. Of the 628 patients, 15.4% were female and 84.6% were male. The median maximum tumor diameter (MTD) was 4.5cm, median percent of patients with portal vein thrombosis (PVT) was present in 37.1% of patients and median serum alpha-fetoprotein (AFP) was 24.3 IU/mL. The serum platelets and liver function tests were either normal (AST) or only slightly abnormal (total bilirubin, ALP, GGT, albumin, platelets) as seen in Table 1, and the median survival of the total unstratified cohort was 38.48+1.35 months, shown in Table 2. However, these median values were not nearly as informative, as in the patient subsets, below.

Survival in patients treated by liver transplant versus other modalities.

One hundred and ninety one patients (30.4% of the cohort) received potentially curative and survival-extending live-donor liver transplants, while 384 patients (61.1% of the cohort) received non-surgical therapies.

The mean and 2-year cumulative survivals are shown in Table 3.

**Figure 1.**
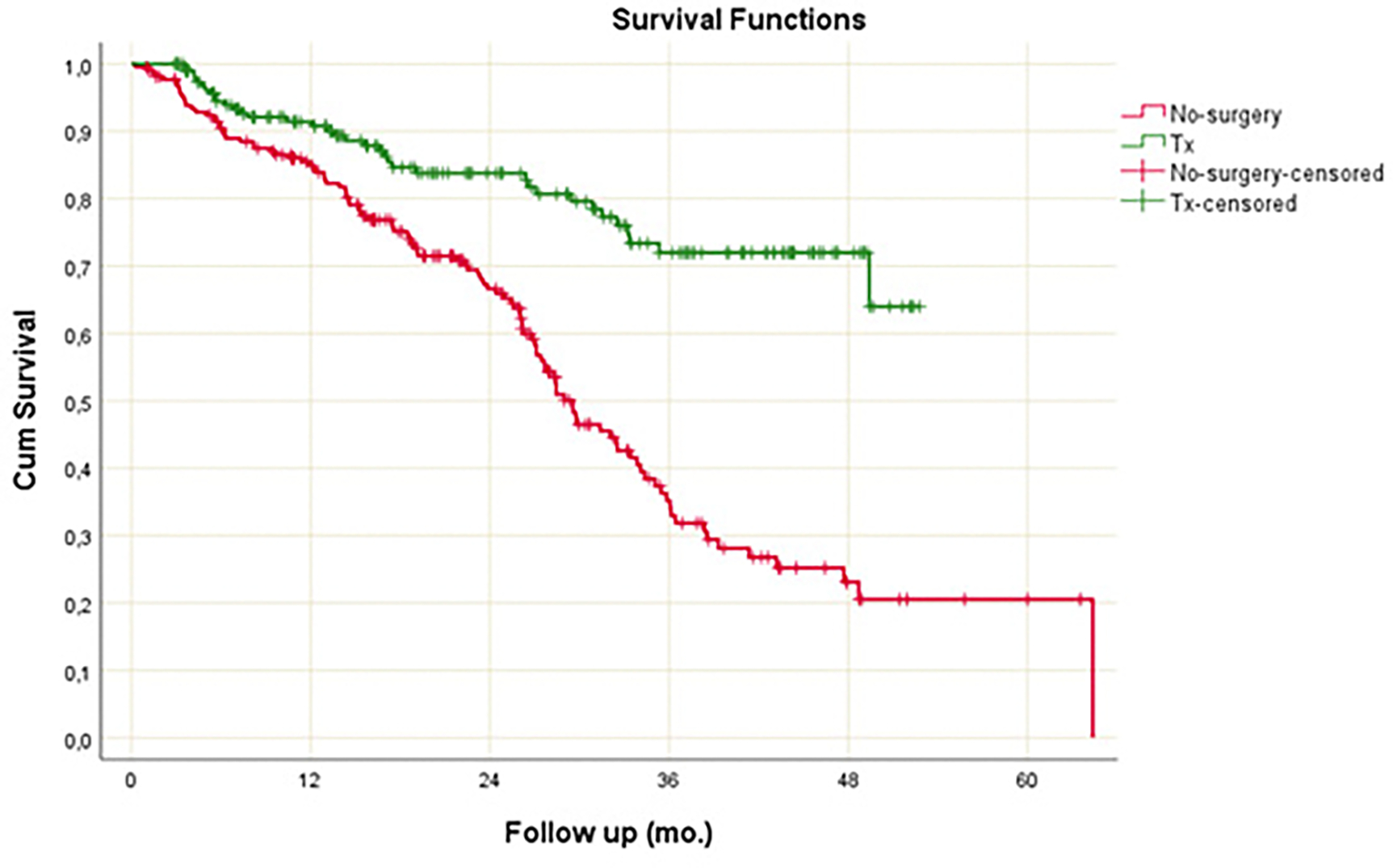
Survival comparison of no surgery and transplant treatment groups.

Transplanted patients had a mean survival of 43.06 + 1.41 months, while non-surgical treated patients had a mean survival of 31.51±1.53 months, p<0.001. The 2-year cumulative survivals were 83.8% and 66.6% respectively, with a hazard ratio (HR) for non-surgery of 3.052 (2.089–4.459, 95% confidence interval, CI) and an HR p-value of <0.001. The cumulative survivals and follow-up are shown in the accompanying Figure 1.

Clinical patient characteristics in transplant and non-transplant treatment groups.

The patient clinical characteristics were next examined, to try to understand the basis for the large survival difference between liver transplantation and non-transplant therapies, shown in Tables 4A and 4B. Maximum tumor diameters (MTDs) were significantly smaller in the transplant compared with the non-transplant group (median MTD was 2.5 cm versus 6.5 cm) and the median serum AFP levels were lower (median 9.6 versus 38.5 IU/mL) and the percent of patients with portal vein thrombosis (PVT) was also much lower (21.3% versus 46.3% of patients) in the transplant versus the non-transplant treatment groups. Serum GGT levels and AFP levels were higher in the non-transplant patients, consistent with their more aggressive tumors. The blood total bilirubin levels were also higher and platelet and albumin levels were lower in the transplanted patients, indicative of more severe cirrhosis. There was significantly more cirrhosis in the transplant compared to the non-transplant group (94.8% versus 40.9%) as well as HBV-based etiology (55% versus 16.1%).

Interestingly, PLR, CRP and ESR levels, which are all indices of inflammation, were higher in the non-transplant group with the larger and more aggressive HCCs. Similarly, large HCCs (>5cm MTD) constituted a significantly higher proportion of the non-transplant versus the transplant patients (58.9% versus 19.7%), as were patients with significantly elevated serum AFP levels >200 IU/mL (38% versus 11.5% patients), significantly elevated serum GGT levels of >150 IU/mL (40.6% versus 19.4% patients) and significantly elevated PLR >150 (36.6% versus 19.9%). By contrast, the number of patients with elevated serum bilirubin levels of >2.0 mg/dL was significantly greater in the transplant group (46.6% versus 17.6% patients).

### Locoregional therapies and best supportive care

Non-metastatic HCC patients in our practice were typically assigned to loco-regional therapies (LRT) of chemoembolization (TACE) previously, or to radioembolization (TARE) in recent years, with a very small proportion who were unsuitable for TARE being referred for oral Sorafenib therapy. Patients with poor performance status or poor liver function who had advanced HCCs and were therefore ineligible for transplant or other anti-cancer modalities, were assigned to best supportive care (BSC), n=53 patients (8.4%). Survival amongst the non-surgery groups is shown in Tables 3 and 5B. Mean survival for loco-regional therapy by transarterial radioembolization (TARE) was 31.51±1.53 months, shown in Table 3. Mean survival for BSC was much lower, at 4.41±0.95 months, shown in Table 5B). There were not enough BSC patients to present 2-year cumulative survival. The subset of HCC patients who had macroscopic portal vein thrombosis (PVT) and who were treated with TARE alone or liver transplant alone, had significant survival differences, with survival after liver transplant alone being much longer, with a mean of 57.89+6.61 months versus 16.01+2.32 months for TARE alone, p=0.001, as shown in Table 5A.

The clinical characteristics of the small BSC group (n=53) were then examined, as shown in Tables 6A and 6B. The tumors were much more aggressive than in any of the other groups, with median MTD of 10cm, PVT in 74.1 percent of patients, median AFP of 636.95 IU/mL, and the liver function was worse than in either the transplant or non-transplant treatment groups, and having median serum GGT levels of 199 IU/mL, and median serum total bilirubin levels of 2.5 mg/dL. Thus, unlike the treated patient groups, the BSC patients had both more aggressive HCC parameters as well as poorer liver function.

## DISCUSSION

HCC is a heterogeneous disease, with variations in incidence, etiology and extent of disease at presentation in different regions of the globe. Furthermore, both the mix of causes (less viral and more metabolic), the stage at diagnosis and the overall survival has been noted to be changing in many countries over the last 30 years [5–7]. The mix of causes has been changing due to the use of effective prevention or treatment of viral causes as well as an increase in obesity (metabolic causes) in many parts of the world. The stage at diagnosis has started to decrease in some countries due to the implementation of active surveillance in patients having predisposing diseases that place them at increased risk for HCC development, such as cirrhosis and its causes. Some countries such as the USA are seeing an increase in survival over time, likely due to earlier diagnosis [5]. Here in Turkiye we have also noticed differences in HCC aggressiveness and survival regionally within the same country [8].

Although most surgical therapy and especially liver transplantation have previously been described as resulting in excellent long-term survival in HCC patients [9, 10], especially compared with non-surgical therapies [11, 12], we were nevertheless interested to examine why so many of our patients were not transplant-eligible. This was not a randomized trial and furthermore, the criteria for liver transplant are well accepted even though they continue to develop [13] and are predominantly based on selection of patients with less aggressive HCCs. The current data is from our weekly liver tumor board and was prospectively collected and from 2019 onwards, the non-surgical and no-treatment (BSC) patient baseline information was also collected. The current study therefore enabled us to capture and compare transplant surgery-treated and non-transplanted HCC patients (who had either non-surgical treatments or no treatments) during the same time period. There were additional patients who returned home to faraway places after their evaluation and whose survival could not be determined. They were thus excluded from this analysis, and so the 628 patients reported here (191 liver transplant, 384 non-surgical oncologic therapies, 53 best supportive care) is to be considered a minimum patient number.

Our principal findings are that just 33.2% of the treated cohort (or 30.4% of the total cohort that included BSC) received a liver transplant and their survival was significantly longer than any other group, as expected. Furthermore, in retrospect they were not the same patients, by virtue of the transplant selection criteria. The liver transplant patients had worse liver function than the non-transplant group and had less aggressive HCCs with smaller tumors, lower serum AFP levels and a much lower percentage of patients with PVT, as shown in Tables 4A and 4B. However, liver failure was a principal treatment aim in liver transplant development.

It has previously been reported that patients with more severe cirrhosis (lower platelet levels as a surrogate marker) have smaller HCCs than patients with less severe cirrhosis, who have higher platelet levels and larger HCCs [14, 15]. There may be at least 2 explanations for this. One is that in the presence of cirrhosis, there may be a limit to the size that an HCC can grow without causing parenchymal liver damage and death from liver failure. A second possible explanation may relate to there being more than one mechanism for HCC growth, with cirrhosis-associated hepatic inflammation being one, and oncogene or growth factor-driven HCC growth being another.

In addition to the liver transplant and non-transplant treatment groups, the third group of BSC patients could not receive any cancer therapy, because their HCCs were too extensive to meet liver transplant criteria plus their poor liver function did not permit safe downstaging to transplant with anti-HCC therapies, in addition to their generally poorer performance status.

Important issues that are raised by these findings concern the reasons why more patients could not be considered for liver transplant, considering the excellent long-term survival that has been reported by many groups for liver transplant within published defined criteria for transplant for HCC. These criteria were developed to include those HCC patients with potential for long-term survival after transplant and to exclude those unlikely to get long-term survival post-transplant, based upon the published experience. The latter group included patients with large tumors, high serum AFP levels and presence of macroscopic PVT.

The ways forward include diagnosing HCC at earlier stages of tumor development (surveillance for those patients with liver diseases placing them at increased risk for HCC development), identification of HCC subgroups that may have better prognosis despite presence of larger tumors or presence of branch PVT, and of treatment of the underlying causes of HCC, including treatment of obesity, hepatitis B and hepatitis C.

Our non-transplant treatment patients were associated with shorter survival than the transplanted patients, as expected. Interestingly, the subgroup of patients with PVT that is traditionally considered to be a poor prognosis marker, had significantly different survivals, depending on whether they were treated with liver transplant alone (57.89±6.61 months) or radioembolization alone (16.01±2.32 months), p=0.001.

Given the very large survival differences between patients who were treated with liver transplant compared with any other patients, it is reasonable to consider that the explanation might not only be the treatment choice, but perhaps a difference in the tumor biology between the 2 treatment groups. The transplanted patients had smaller HCCs (by choice of treatment selection) and therefore may have had slower-growing tumors with less aggressive biology, to explain why their tumors were smaller. On this view, the patients with larger tumors had more aggressive tumor biology (accompanied by higher serum AFP levels and increased percent of patients with PVT) and their shorter survival may thus have been predicated on their more aggressive tumor biology, which resulted in those patients having tumors that grew beyond the transplant criteria. The treatment and biology can therefore be viewed in 2 opposite ways, namely that their transplant caused them to have a longer survival, or contrariwise, they had better tumor biology, resulting in their having longer survival after transplant.

There are some newer approaches being considered. Firstly, we continue to interrogate our expanding HCC database to try retrospectively to identify characteristics of patient subsets with longer survival, which can then be applied to future therapy selection. Secondly, this approach is currently being applied to patients with macroscopic PVT to attempt to identify future patient subsets who might benefit from transplantation and longer survival, despite the presence of PVT. In this regard, neo-adjuvant TARE and stereotactic body radiotherapy (SBRT) are being evaluated. Thirdly, we are considering longer cancer treatment courses and more aggressive neo-adjuvant therapies, to decrease elevated levels of the HCC biomarkers AFP and GGT, to determine if that permits subsequent transplantation with prolonged survival, especially in those patients with baseline AFP levels >1,000 IU/mL which can potentially be substantially decreased pre-transplant. Fourthly, we are starting to implement a Network Phenotyping Strategy, to try to identify at baseline, patients who might have better prognostic characteristics [16]. Ultimately however, the roles of prevention and earlier HCC diagnosis will have the greatest impact of all [17], especially since only 30.4% of our total cohort qualified for liver transplant, as the remaining patients had either advanced stage HCC at presentation, that precluded liver transplant under current guidelines, or had poor liver function that precluded pre-transplant oncologic therapy. We therefore need more systematic surveillance of patients at risk for HCC development, and more aggressive HCC downstaging by oncologic therapy pre-transplant. We might also consider including for liver transplant in the future, some patients with larger size HCCs and that subset of HCC patients with PVT who have favorable biomarker characteristics. At the time of this writing, the longest reported non-transplant survival for combination immune checkpoint inhibitor therapy is 19.6% at 5-years for durvalumab plus tremelimumab, the STRIDE regimen [18], which is still a long way from the minimum of 75% 5-year survival that can typically be achieved by liver transplant, for HCC patients who are within current guidelines. The cost of STRIDE therapy is reportedly $46,000 for the first cycle and then approximately $12,000 for subsequent cycles [19]. Thus, over 2 years, the cost of STRIDE and liver transplant at our institution ($200,000) are similar, with one of them (transplant) being potentially curative and the other being continued till tumor progression. Since our program is based on live donor liver transplantation, usually from family members of the patient, there is no competition for these same organs, if liver transplant for patients with more advanced HCC is to be considered. Immune checkpoint inhibitors are offered for HCC patients beyond transplant criteria in many countries, and are also being evaluated for use in the pre-transplant neo-adjuvant setting. Since their current use is for the first line of systemic treatment of HCC patients who are beyond current transplant criteria, perhaps their combination with liver transplant in patients with more advance HCC might be a reasonable future step.

Limitations of this study include the relatively small patient numbers in each of the 3 groups and the fact that the patient groups were dissimilar. Furthermore, the non-transplant treated patients often went on to other therapies after failing LRT, as is standard oncological practice.

### Conclusions.

We are currently able to offer potentially curative liver transplantation to only 30% of the HCC patients who present to us with newly diagnosed HCC, because of advanced disease at presentation in the other 70% of patients. A multipronged approach is feasible to what could lead to improved survival in this multifaceted disease.

## Figures and Tables

**Table 1.** Clinical characteristics of unstratified total cohort [n=628].

	Median [IQR]	[Min.-Max.]
Age	61 [13]	[1–89]
MTD [cm]	4.5 [6.5]	[0–35]
AFP	24.3 [393.5]	[0–97248]
Neutros	3.65 [2.9]	[0.42–48.8]
Lymphs	1.27 [0.9]	[0–18]
Platelets	144 [139]	[2.64–920]
PLR	111.34 [107.13]	[1.66–6423.08]
Albumin	3.3 [1.1]	[1–25]
T.Bil	1.34 [1.52]	[0–33.83]
AST	57 [63]	[12–7789]
ALKP	134 [110]	[21–2327]
GGT	105 [154]	[8–1620]
CRP	0.93 [2.32]	[0–351]
ESR	23 [33]	[0–119]

Abbreviations: HCC, hepatocellular carcinoma; WBC, White blood count; TBil, Total bilirubin; AST, Aspartate amino transferase; ALKP, Alkaline phosphatase; GGT, Gamma glutamyl transferase; neutron, neutrophils; Lymphs, lymphocytes; MTD, maximum tumor diameter; PVT, portal vein thrombosis; AFP, alpha-fetoprotein; CRP, C-reactive protein; ESR, erythrocyte sedimentation rate.

**Table 2.** Survival of unstratified total cohort.

Survival [mo.] Mean±SE	2-years cumulative survival rate
38.48±1.35	70.3%

**Table 3.** Survival comparison between treatment groups, non-transplant or liver transplant patients.

	Survival [mo.] Mean±SE	Log-Rank p-value	2-years cumulative survival rate	HR [95% C.I.]	HR p-value

No					
Tx[n=384]	31.51±1.53	<0.001	62.6%	3.052 [2.089–4.459]	<0.001
Tx [n=191]	43.06±1.41		83.8%	reference	

**Table 4A.** Clinical characteristics of non-transplant and liver transplant treatment groups.

	Non-transplant [n=384]		Transplant [n=191]		
Parameter	Median [IQR]	[Min.-Max.]	Median [IQR]	[Min.-Max.]	p
Age	63 [13]	[18–89]	57 [15]	[1–72]	<0.001
MTD [cm]	7 [8.9]	[0.4–35]	2.5 [3.23]	[0–26]	<0.001
AFP	53.05 [994.71]	[0–97248]	9.6 [34.1]	[0.3–55000]	<0.001
Neutros	4 [3.18]	[0.42–48.8]	3 [2.27]	[0.68–22.9]	<0.001
Lymphs	1.32[0.88]	[0–8.59]	1.11 [0.88]	[0.03–18]	<0.001
Platelets	174.5 [145.25]	[2.64–920]	95 [83]	[15–701]	<0.001
PLR	125.27 [102.59]	[1.66–1002.86]	81.48 [70.68]	[2.61–6423.08]	<0.001
Albumin	3.4 [1.1]	[1–25]	3.1 [1]	[1.5–5.2]	<0.001
T.Bil	1.17 [1.3]	[0–33.83]	1.9 [2.73]	[0.23–32.7]	<0.001
AST	59 [66]	[12–782]	57 [57]	[14–7789]	0.732
ALP	139.5 [128.25]	[21–1649]	121 [91]	[36–2327]	0.023
GGT	130.5 [190.25]	[8–1620]	77 [86]	[12–702]	<0.001
CRP	1.17 [3.47]	[0–351]	0.83 [1.37]	[0.3–41.7]	0.003
ESR	29 [34]	[0–119]	21 [31.5]	[0–109]	0.018

Abbreviations: PLR, platelet lymphocyte ratio; HBV, hepatitis B virus; HCV, hepatitis C virus; others as in Table 1.

**Table 4B.** Clinical Characteristics of non-transplant and transplant treatment groups [cont].

Parameter [%]		Non-transplant [n=384]	Transplant [n=191]	p
Gender	Female	18	10.5	0.026
	Male	82	89.5	
Cirrhosis	No	59.1	5.2	<0.001
	Yes	40.9	94.8	
HBV	No	83.9	45	<0.001
	Yes	16.1	55	
HCV	No	95.8	91.6	0.060
	Yes	4.2	8.4	
PVT	No	53.7	78.7	<0.001
	Yes	46.3	21.3	
Number of nodules	1	48.9	46.8	0.659
	>1	51.1	53.2	
MTD	≤5 cm	41.1	80.3	<0.001
	>5 cm	58.9	19.7	
AFP	≤200	62	88.5	<0.001
	>200	38	11.5	
GGT	≤150	59.4	80.6	<0.001
	>150	40.6	19.4	
T.Bil	≤2	82.4	53.4	<0.001
	>2	17.6	46.6	
PLR	≤150	63.4	80.1	<0.001
	>150	36.6	19.9	

**Table 5A.** Survival comparison between treatment groups in PVT positive patients.

	Survival [mo.] Mean±SE	Log-Rank p-value	2-years cumulative survival rate	HR [95% C.I.]	HR p-value
TARE					
[n=27]	16.01±2.32	0.001	35.3%	2.653 [1.422–4.950]	0.002
Tx [n=61]	57.89±6.61		64.8%	reference	

Abbreviations: TARE, transarterial radioembolization; Tx, liver transplantation.

**Table 5B.** Survival of BSC, no-treatment patients.

	Survival [mo.] Mean±SE	Survival [mo.] Median±SE
BSC [n=53]	4.41±0.95	1.5±0.28

BSC, best supportive care.

**Table 6A.** Clinical Characteristics of BSC group [n=53].

BSC		
	Median [IQR]	[Min.-Max.]
Age	63 [10]	[27–84]
MTD [cm]	10 [14.4]	[1.8–25]
AFP	636.95 [1709]	[0.9–97248]
Neutros	4.8 [4.4]	[1.6–25.3]
Lymphs	1.2 [0.59]	[0.3–4.2]
Platelets	168.5 [142.25]	[11.2–405]
PLR	135.01 [105.56]	[16–876.67]
Albumin	2.9 [0.85]	[1.7–25]
T.Bil	2.5 [4.58]	[0.5–27.1]
AST	125 [137.75]	[28–397]
GGT	199 [295.5]	[26–1179]

Abbreviations: BSC, best supportive care; others, as in Table 1.

**Table 6B.** Clinical Characteristics of BSC group [cont].

Parameter [%]	BSC	
Gender	Female	10.9
	Male	89.1
Cirrhosis	No	43.9
	Yes	56.1
HBV	No	84.2
	Yes	15.8
HCV	No	98.2
	Yes	1.8
PVT	No	25.9
	Yes	74.1
Number of nodules	1	35.7
	>1	64.3
MTD	≤5 cm	25.9
	>5 cm	74.1
AFP	≤200	34.6
	>200	65.4
GGT	≤150	35.8
	>150	64.2
T.Bil	≤2	37.7
	>2	62.3
PLR	≤150	54.7
	>150	45.3

Abbreviations: as in Table 1.

## Abbreviations

HCC: hepatocellular carcinoma
WBC: White blood count
TBil: Total bilirubin
AST: Aspartate amino transferase
ALKP: Alkaline phosphatase
GGT: Gamma glutamyl transferase
MTD: maximum tumor diameter
PVT: portal vein thrombosis
AFP: alpha-fetoprotein
CAT: computed axial tomography
PLR: platelet lymphocyte ratio
HR: hazard ratio
HBV: hepatitis B virus
HCV: hepatitis C virus CRP, C-reactive protein
ESR: erythrocyte sedimentation rate
Tx: transplant
LRT: loco-regional therapy
TARE: transarterial radioembolization
TACE: transarterial chemoembolization
SBRT: stereotactic body radiation therapy
BSC: best supportive care

## References

[1] Sankar K, Gong J, Osipov A, Miles SA, Kosari K, Nissen NN, Hendifar AE, Koltsova EK, Yang JD. Recent advances in the management of hepatocellular carcinoma. Clin Mol Hepatol. 2024 Jan;30[1]:1–15. doi: 10.3350/cmh.2023.0125. Epub 2023 Jul 21. 37482076 PMC10776289

[2] Finn RS, Qin S, Ikeda M, Galle PR, Ducreux M, Kim TY, Kudo M, Breder V, Merle P, Kaseb AO, Atezolizumab plus Bevacizumab in Unresectable Hepatocellular Carcinoma. N. Engl. J. Med. 2020;382:1894–1905. doi: 10.1056/NEJMoa1915745. 32402160

[3] Wen F, Huang P, Wu Q, Yang Y, Zhou K, Zhang M, Li Q. Promising first-line immuno-combination therapies for unresectable hepatocellular carcinoma: A cost-effectiveness analysis. Cancer Med. 2024 Aug;13[16]:e70094. doi: 10.1002/cam4.70094. 39149756 PMC11327610

[4] Sangro B, Chan SL, Kelley RK, Lau G, Kudo M, Sukeepaisarnjaroen W, Yarchoan M, De Toni EN, Furuse J, Kang YK, Galle PR, Rimassa L, Heurgué A, Tam VC, Van Dao T, Thungappa SC, Breder V, Ostapenko Y, Reig M, Makowsky M, Paskow MJ, Gupta C, Kurland JF, Negro A, Abou-Alfa GK; HIMALAYA investigators. Four-year overall survival update from the phase III HIMALAYA study of tremelimumab plus durvalumab in unresectable hepatocellular carcinoma. Ann Oncol. 2024 May;35[5]:448–457. doi: 10.1016/j.annonc.2024.02.005. Epub 2024 Feb 19. 38382875

[5] American Cancer Society. Cancer Facts & Figures 2024. Atlanta: American Cancer Society; 2024.

[6] Siegel RL, Giaquinto AN, Jemal A. Cancer statistics, 2024. CA Cancer J Clin. 2024 Jan-Feb;74[1]:12–49. doi: 10.3322/caac.21820. Epub 2024 Jan 17. Erratum in: CA Cancer J Clin. 2024 Mar-Apr;74[2]:203. doi: 10.3322/caac.21830. 38230766

[7] Rumgay H, Arnold M, Ferlay J, Lesi O, Cabasag CJ. Global burden of primary liver cancer in 2020 and predictions to 2040. Journal of Hepatology 2022 ;77 :1598–1606 . 36208844 10.1016/j.jhep.2022.08.021 PMC9670241

[8] Akkiz H, Carr BI, Yalçın KK, Guerra V, Kuran S, Altıntaş E, Üsküdar O, Karaoğullarından Ü, Özakyol A, Tokmak S, Yücesoy M, Bahçeci Hİ, Ülkü A, Akçam T, Yalçın Polat K, Ekinci N, Şimşek H, Örmeci N, Sonsuz A, Demir M, Kılıç M, Uygun A, Ballı T, Demir A, Arslan B, Doran F. Characteristics of Hepatocellular Carcinoma Aggressiveness Factors in Turkish Patients. Oncology. 2018;94[2]:116–124. doi: 10.1159/000484564. Epub 2017 Dec 6. 29207378 PMC5828952

[9] Mehta N, Bhangui P, Yao FY, Mazzaferro V, Toso C, Akamatsu N, Durand F, Ijzermans J, Polak W, Zheng S, Roberts JP, Sapisochin G, Hibi T, Kwan NM, Ghobrial M, Soin A. Liver Transplantation for Hepatocellular Carcinoma. Working Group Report from the ILTS Transplant Oncology Consensus Conference. Transplantation. 2020 Jun;104[6]:1136–1142. doi: 10.1097/TP.0000000000003174. 32217938

[10] Tabrizian P, Holzner ML, Mehta N, Halazun K, Agopian VG, Yao F, Busuttil RW, Roberts J, Emond JC, Samstein B, Brown RS Jr, Najjar M, Chapman WC, Doyle MM, Florman SS, Schwartz ME, Llovet JM. Ten-Year Outcomes of Liver Transplant and Downstaging for Hepatocellular Carcinoma. JAMA Surg. 2022 Sep 1;157[9]:779–788. doi: 10.1001/jamasurg.2022.2800. 35857294 PMC9301590

[11] Shin SW, Ahn KS, Kim SW, Kim TS, Kim YH, Kang KJ. Liver Resection Versus Local Ablation Therapies for Hepatocellular Carcinoma Within the Milan Criteria: A Systematic Review and Meta-analysis. Ann Surg. 2021 Apr 1;273[4]:656–666. doi: 10.1097/SLA.0000000000004350. 33074898

[12] Bogdanovic A, Djokic Kovac J, Zdujic P, Djindjic U, Dugalic V. Liver resection versus transarterial chemoembolisation for the treatment of intermediate hepatocellular carcinoma: a systematic review and meta-analysis. Int J Surg. 2023 May 1;109[5]:1439–1446. doi: 10.1097/JS9.0000000000000344. 37222718 PMC10389385

[13] Ince V, Akbulut S, Otan E, Ersan V, Karakas S, Sahin TT, Carr BI, Baskiran A, Samdanci E, Bag HG, Koc C, Usta S, Ozdemir F, Barut B, Gonultas F, Sarici B, Kutluturk K, Dogan MS, Ozgor D, Dikilitas M, Harputluoglu M, Aladag M, Kutlu R, Varol I, Dirican A, Aydin C, Isik B, Ara C, Kayaalp C, Emre S, Yilmaz S. Liver Transplantation for Hepatocellular Carcinoma: Malatya Experience and Proposals for Expanded Criteria. J Gastrointest Cancer. 2020 Sep;51[3]:998–1005. doi: 10.1007/s12029-020-00424-w. Erratum in: J Gastrointest Cancer. 2020 Sep;51[3]:1006. doi: 10.1007/s12029-020-00451-7. 32519232

[14] Carr BI, Bag HG, Yilmaz S. Peripheral blood platelet counts identify prognostically diverse clinical phenotypes in hepatocellular carcinoma. Ann Gastroenterol Dig Syst. 2024;7[1]:1081. Epub 2024 May 13. 38887309 PMC11182490

[15] Carr BI, Guerra V, Giannini EG, Farinati F, Ciccarese F, Rapaccini GL, Di Marco M, Benvegnù L, Zoli M, Borzio F, Caturelli E, Chiaramonte M, Trevisani F; Italian Liver Cancer Group. Significance of platelet and AFP levels and liver function parameters for HCC size and survival. Int J Biol Markers. 2014 Sep 30;29[3]:e215–23. doi: 10.5301/jbm.5000064. 24526315

[16] Pančoška P, Skála L, Nešetřil J, Carr BI. Validation of the Concept of a Common Typical Time of Disease Duration for Hepatocellular Carcinoma Patients Using the Fisher Information Processing of Tumor Imaging Results Combined with Network Phenotyping Strategy Quantification of Individual Patient Clinical Profile Patterns. Semin Oncol. 2015 Aug;42[4]:672–8. doi: 10.1053/j.seminoncol.2015.05.004. Epub 2015 Jun 3. 26320070

[17] Yilma M, Houhong Xu R, Saxena V, Muzzin M, Tucker LY, Lee J, Mehta N, Mukhtar N. Survival Outcomes Among Patients with Hepatocellular Carcinoma in a Large Integrated US Health System. JAMA Netw Open. 2024 Sep 3;7[9]:e2435066. doi: 10.1001/jamanetworkopen.2024.35066. 39316399 PMC11423175

[18] Rimassa L, Chan SL, Sangro B Annals of Oncology 2024; 35: S656–S673 [ESMO].

[19] Liao W, Xu H, Hutton D, Wu Q, Yang Y, Feng M, Lei W, Bai L, Li J, Li Q. Cost-effectiveness analysis of durvalumab plus tremelimumab as first-line therapy in patients with unresectable hepatocellular carcinoma. Ther Adv Med Oncol. 2024 Sep 18;16:17588359241274625. doi: 10.1177/17588359241274625. 39301138 PMC11412210

